# Supplementary figures and images for: BioPETsurv: Methodology and open source software to evaluate biomarkers for prognostic enrichment of time-to-event clinical trials
Source: PLoS One. 2020 Sep 18;15(9):e0239486. doi: 10.1371/journal.pone.0239486 (PMC7500596; doi:10.1371/journal.pone.0239486)

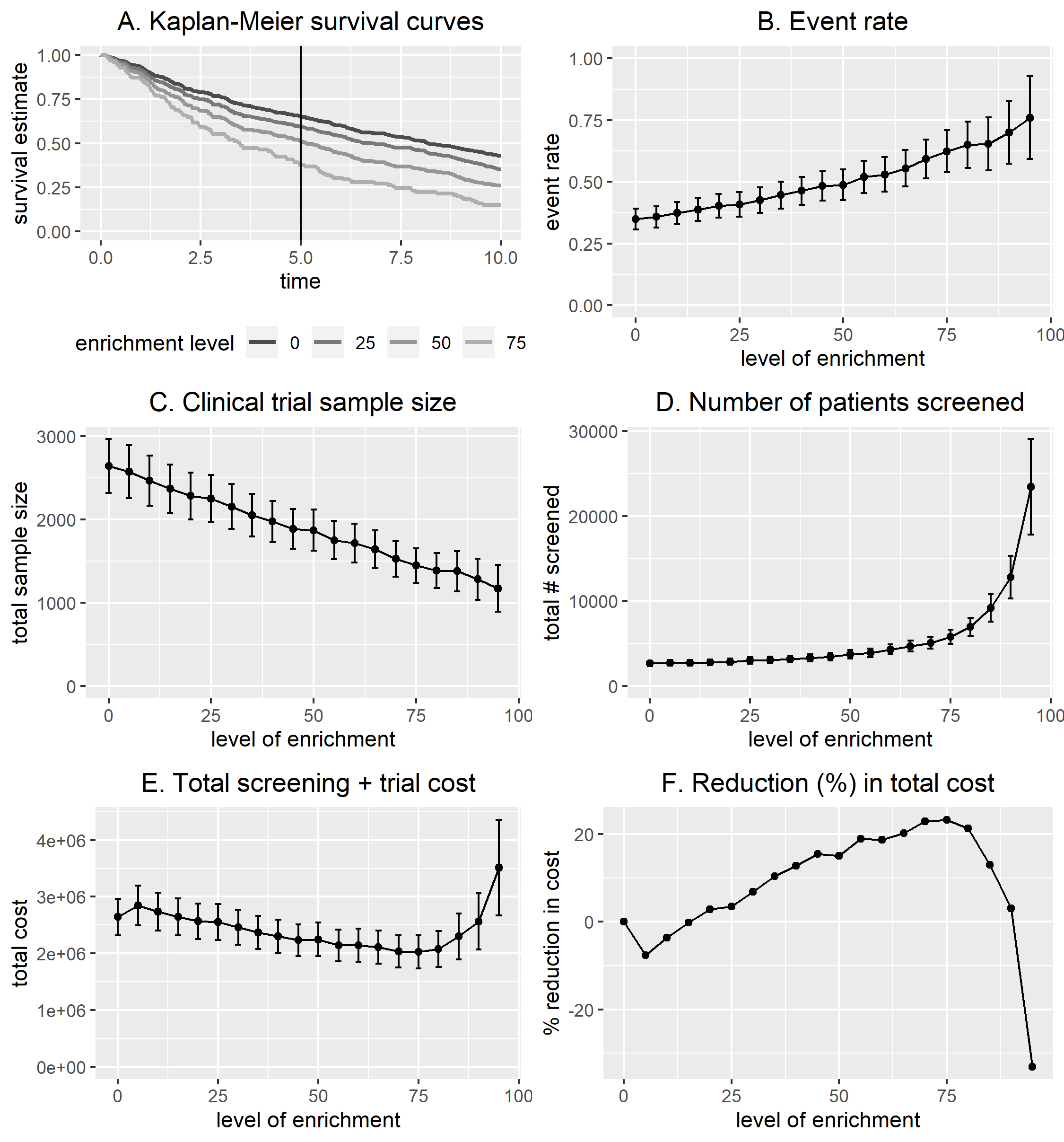

Supplement: S1 Fig — (TIF) [file pone.0239486.s001.tif]

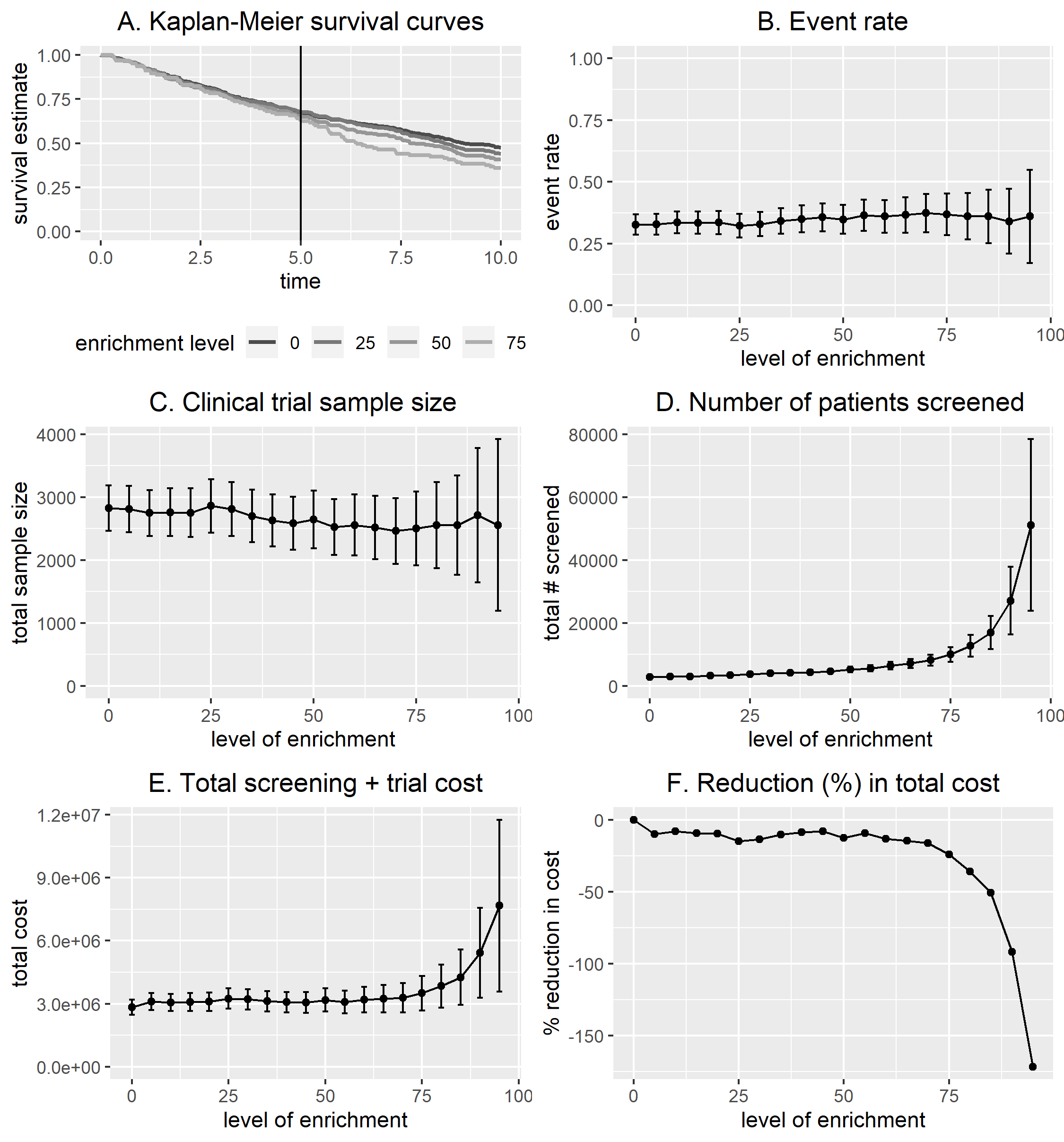

Supplement: S2 Fig — (TIF) [file pone.0239486.s002.tif]
