# Supplementary material for: BioPETsurv: Methodology and open source software to evaluate biomarkers for prognostic enrichment of time-to-event clinical trials
Source: PLoS One. 2020 Sep 18;15(9):e0239486. doi: 10.1371/journal.pone.0239486 (PMC7500596; doi:10.1371/journal.pone.0239486)
Supplement: S1 Table — (DOCX) [file pone.0239486.s003.docx]

**Table S1:**  **BioPETsurv analysis of a highly prognostic biomarker (simulated) for a fixed-duration 5-year trial.**

We used the BioPETsurv data simulator and specified constant baseline hazard function, 50% survival at 10 years, and biomarker hazard ratio 2.0 per SD of the marker. We further specified a fixed-duration 5-year trial and 90% power to detect a treatment hazard ratio of 0.8 using two-sided hypothesis testing at α-level 0.05. With $100/patient screening cost and $1000/patient trial cost, total costs are minimized at the 75% level of enrichment, with about 23% cost reduction over an unenriched trial. Results are also shown in Figure S1.

| **Screening Threshold** | **Event Rate (%)** | **Sample Size** | **Total Screened** | **Reduction in Total Cost (%)** |
| --- | --- | --- | --- | --- |
| **0%** | 35 | 2642 | 2642 | 0 |
| **5%** | 36 | 2573 | 2709 | -7.6 |
| **10%** | 37 | 2464 | 2738 | -3.6 |
| **15%** | 39 | 2367 | 2785 | -0.1 |
| **20%** | 40 | 2281 | 2852 | 2.9 |
| **25%** | 41 | 2250 | 3000 | 3.5 |
| **30%** | 43 | 2154 | 3078 | 6.8 |
| **35%** | 45 | 2052 | 3157 | 10.4 |
| **40%** | 46 | 1974 | 3290 | 12.8 |
| **45%** | 48 | 1888 | 3433 | 15.6 |
| **50%** | 49 | 1871 | 3742 | 15.0 |
| **55%** | 52 | 1752 | 3894 | 19.0 |
| **60%** | 53 | 1717 | 4293 | 18.8 |
| **65%** | 55 | 1639 | 4683 | 20.2 |
| **70%** | 59 | 1526 | 5087 | 23.0 |
| **75%** | 62 | 1447 | 5788 | 23.3 |
| **80%** | 65 | 1386 | 6931 | 21.3 |
| **85%** | 65 | 1379 | 9194 | 13.0 |
| **90%** | 70 | 1281 | 12811 | 3.0 |
