# Supplementary material for: BioPETsurv: Methodology and open source software to evaluate biomarkers for prognostic enrichment of time-to-event clinical trials
Source: PLoS One. 2020 Sep 18;15(9):e0239486. doi: 10.1371/journal.pone.0239486 (PMC7500596; doi:10.1371/journal.pone.0239486)
Supplement: S2 Table — (DOCX) [file pone.0239486.s004.docx]

**Table S2:**  **BioPETsurv analysis of a weakly prognostic biomarker (simulated) for a fixed-duration 5-year trial.**

Specifications are identical to S1 except that the biomarker hazard ratio is 1.2 per SD of the marker. With $100/patient screening cost and $1000/patient trial cost, an enriched trial is more expensive than an unenriched trial for any level of enrichment. Results are also shown in Figure S2.

| **Screening Threshold** | **Event Rate (%)** | **Sample Size** | **Total Screened** | **Reduction in Total Cost (%)** |
| --- | --- | --- | --- | --- |
| **0%** | 33 | 2824 | 2824 | 0 |
| **5%** | 33 | 2809 | 2957 | -9.9 |
| **10%** | 34 | 2748 | 3054 | -8.1 |
| **15%** | 33 | 2760 | 3248 | -9.2 |
| **20%** | 34 | 2752 | 3440 | -9.6 |
| **25%** | 32 | 2860 | 3814 | -14.8 |
| **30%** | 33 | 2807 | 4011 | -13.6 |
| **35%** | 34 | 2699 | 4153 | -10.3 |
| **40%** | 35 | 2632 | 4387 | -8.7 |
| **45%** | 36 | 2584 | 4699 | -8.1 |
| **50%** | 35 | 2647 | 5294 | -12.5 |
| **55%** | 36 | 2526 | 5614 | -9.3 |
| **60%** | 36 | 2557 | 6393 | -13.2 |
| **65%** | 37 | 2517 | 7192 | -14.6 |
| **70%** | 37 | 2464 | 8214 | -16.3 |
| **75%** | 37 | 2501 | 10004 | -24.0 |
| **80%** | 36 | 2557 | 12786 | -35.8 |
| **85%** | 36 | 2557 | 17047 | -50.9 |
| **90%** | 34 | 2711 | 27111 | -92.0 |
