# Supplementary material for: BioPETsurv: Methodology and open source software to evaluate biomarkers for prognostic enrichment of time-to-event clinical trials
Source: PLoS One. 2020 Sep 18;15(9):e0239486. doi: 10.1371/journal.pone.0239486 (PMC7500596; doi:10.1371/journal.pone.0239486)
Supplement: Attachment — Submitted filename: PONE-D-20-12411-report.pdf [file pone.0239486.s005.pdf]

Referee Report
PLOS ONE
PONE-D-20-12411
BioPETsurv: Methodology and open source softwre to evaluate biomarkers for prognostic enrichment of time-to-event clinical trials

Authors developed an R package BioPETsurv (released in January 2020) and an R shiny app available at `http://prognosticenrichment.com/surv/` as a biomarker prognostic enrichment tool for survival analysis. The R package BioPETsurv is an extension of another package BioPET (released in July 2018). With these tools, clinical trialists can develop a protocol on a trial with prognostic enrichment faster and more effectively. This package also allows investigators to explore prognostic enrichment with simulated data. The total cost is computed as a linear combination of the cost for a patient in the trial and that of screening. The sample size estimations for fixed-duration trials and trials with an accrual period and a follow-up period follow the formulas in Schoenfeld (1983, Biometrics), *Sample-size formula for the proportional-hazards regression model.* This manuscript is well-structured and well-written, and the documents for the R package and shiny app are also well-written. I only have a few comments below.

Comments

- Under the settings in Table 1 and 2, the Reduction in Total Cost is generally an inceasing function of the Screening Threshold. Authors can add additional situation where the Reduction in Total Cost is not a monotonic function of the Screening Threshold. For example, with effect size 2, Cost of screening a patient to determine trial eligibility 100 and Cost of running a patient through the trial 100, the maximum Reduction in Total Cost is achieved around Screening Threshold = 75%.

- Authors can discuss the general relation between the Reduction in Total Cost

and the Screening Threshold. For example, when the relation is monotonic, when it is not.

- The resolution of Figure 1 and 2 is quite low. I guess the reason is that the ggplot figures were first imbedded into MS Word and then were converted to pdf. Please make sure to have high resolution images for the final submission.
- There is a typo on the shiny app: "eligiblity" should be spelled as "eligibility".
