# Supplementary material for: BioPETsurv: Methodology and open source software to evaluate biomarkers for prognostic enrichment of time-to-event clinical trials
Source: PLoS One. 2020 Sep 18;15(9):e0239486. doi: 10.1371/journal.pone.0239486 (PMC7500596; doi:10.1371/journal.pone.0239486)
Supplement: Attachment — Submitted filename: PONE-D-20-12411_R2-report.pdf [file pone.0239486.s009.pdf]

Referee Report
PLOS ONE
PONE-D-20-12411R2
BioPETsurv: Methodology and open source softwre to evaluate biomarkers for prognostic enrichment of time-to-event clinical trials

- Authors have added two tables and two figures in supplementary material for the comment: "Under the settings in Table 1 and 2, the Reduction in Total Cost is generally an increasing function of the Screening Threshold. Authors can add additional situation where the Reduction in Total Cost is not a monotonic function of the Screening Threshold. For example, with effect size 2, Cost of screening a patient to determine trial eligibility 100 and Cost of running a patient through the trial 100, the maximum Reduction in Total Cost is achieved around Screening Threshold = 75%." I have no additional comment.
