# Supplementary material for: BioPETsurv: Methodology and open source software to evaluate biomarkers for prognostic enrichment of time-to-event clinical trials
Source: PLoS One. 2020 Sep 18;15(9):e0239486. doi: 10.1371/journal.pone.0239486 (PMC7500596; doi:10.1371/journal.pone.0239486)
Supplement: Attachment — Submitted filename: ReviewResponse.docx [file pone.0239486.s008.docx]

**Reviewers' comments**

Reviewer #1 and #2 agreed that our article is technically sound piece of research with data that support the conclusions, and that our analysis is appropriate and rigorous. Reviewer #1 had one additional comment:

*Reviewer #1: Authors have edited this manuscript successfully. I only have one comment.*

*Authors have added additional descriptions in Discussion for the previous comment: “Under the settings in Table 1 and 2, the Reduction in Total Cost is generally an increasing function of the Screening Threshold. Authors can add additional situation where the Reduction in Total Cost is not a monotonic function of the Screening Threshold. For example, with effect size 2, Cost of screening a patient to determine trial eligibility 100 and Cost of running a patient through the trial 100, the maximum Reduction in Total Cost is achieved around Screening Threshold = 75%.” In order to keep the manuscript succinct, authors decided not to include additional illustrations. However, readers will be benefited from these additional illustrations. Therefore, authors may include these part into supplement material.*

Response: Our revised submission includes Supplementary Tables S1 and S2 and Supplementary Figures S1 and S2 to illustrate these additional situations.
